# Haze of glue determines preference of western flower thrips (*Frankliniella occidentalis*) for yellow or blue traps

**DOI:** 10.1038/s41598-021-86105-5

**Published:** 2021-03-22

**Authors:** Robert W. H. M. van Tol, Jolanda Tom, Monika Roher, Anne Schreurs, Coby van Dooremalen

**Affiliations:** 1 Wageningen University and Research, P.O. Box 69, 6700 AB Wageningen, The Netherlands; 2 Faculty of Agriculture, University of Zagreb, Svetošimunska cesta 25, 10000 Zagreb, Croatia; 3 HAS University of Applied Sciences, Onderwijsboulevard 221, 5223 DE ’s-Hertogenbosch, The Netherlands

**Keywords:** Plant sciences, Optics and photonics

## Abstract

In a wind tunnel we compared the colour preference for western flower thrips to four types of colour plates (clear, white, blue and yellow) applied with two types of glue (diffuse Stikem versus clear D41). Further the results for blue and yellow preference were validated in two greenhouses. In the wind tunnel, we found a clear preference of yellow over blue when a clear glue (D41) was used. However, with a more diffuse (whitish) glue (Stikem) the preference for yellow over blue disappeared, whereby the attraction to yellow decreased (58%) while the attraction to blue increased (65%). In the greenhouses, we found similar effects as in the wind tunnel with a decrease in attraction to yellow (35%) and increase in attraction to blue (32%) for Stikem compared to D41. Light measurements showed an increase of 18% of blue, 21% of violet light, 8% of yellow and 9% of green light reflected on the yellow Stikem trap versus the yellow D41 trap. On blue plates there was only 4% increase of blue light, 8% decrease of yellow light reflected when Stikem glue was used compared to D41 glue. It is not yet clear if the change of light reflection ratio blue/yellow caused by the glue type plays a role in the change of attraction. The reflective properties of glue are so far an unknown factor in colour choice and may explain partially the different results on colour preference. A small review on thrips colour preference is discussed to determine possible other factors of influence on colour choice.

## Introduction

Western flower thrips, *Frankliniella occidentalis* Pergande (Thysanoptera: Thripidae) is considered to be one of the most damaging insect species in greenhouses worldwide. It is a polyphagous pest with a short life cycle and high fecundity^1,2^ causing direct damage (e.g. malformation and discolouration) to leaves, flowers and fruit, economic damage (through quarantine) and it is an important vector of tospoviruses^3,4^. Western flower thrips is not only damaging but also difficult to control due to its cryptic life cycle^3,5^. The rapid resistance to different insecticide classes^6–11^, makes it a difficult species to control^12^.

Visual attractive sticky plate combined with attractants^13,14^ are the most widely used and effective tools to monitor western flower thrips. The attractant aids in bringing the thrips close to the trap, when visual detection takes over and leads to increased landing on these traps. The preferred colour of the trap for western flower thrips has long been discussed and mainly three colours are mentioned as effective for trapping, namely blue, yellow and white. The results on the attraction to these colours are very variable between locations and the factors contributing to this variability in attraction apart from colour is unclear. Matteson et al.^15^ is the only electrophysiological study on *F. occidentalis* compound eye and visual perception performed. They found spectral efficiency peaks in the ultraviolet and the green range via electroretinogram recordings. However, most publications mention blue^4,16–23^, some yellow^24,25^ and occasionally white/blue^26,28^ as the most attractive colour in comparative field and greenhouse studies. The attraction to blue is different from what expected according to this study and has not been explained so far. Colour opponency is well established for e.g. honey bees, bumble bees and other insects, including several herbivores like *F. occidentalis*^29^ where addition of green to blue and vice versa reduces attraction and for whitefly where small amounts of blue reduces attraction to green^30^. This opponency mechanism suggests that the output of several photoreceptors cause an antagonistic neural interaction^29,31–33^. This may explain differential colour preference found.

**Figure 1:**
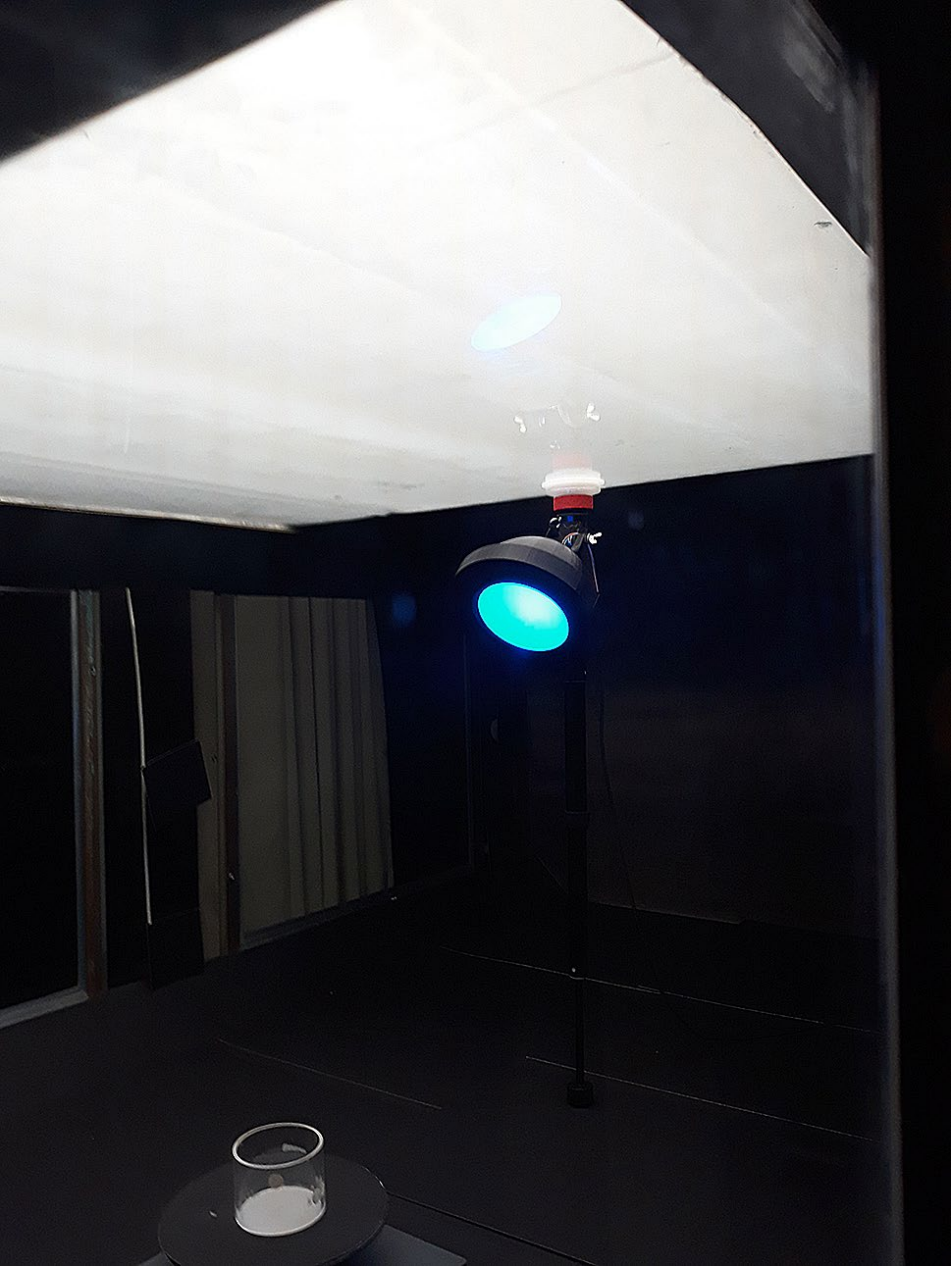
Wind tunnel setting of LED testing. The roof light (white top) is prevented from reflecting light on the glass plate of the cone (yellow LED shining through the glass plate) by facing 45° downwards.

Temperature, humidity, life stage, migration, sexes, greenhouse, climate, light and many other factors are also suggested to play a role in the colour attraction but never proven. A neglected factor also has been the visual properties of glue on these sticky plates. The idea of glue as a factor came to our knowledge when we were testing the effect of different LED colours shining through a light diffusing glass plate. In a wind tunnel with roof light simulating sunlight the glass plate with glue appeared to repel western flower thrips when the roof light was reflecting on the plates facing 45° up despite a coloured LED light shining from behind through the plate. While if the glass plate was facing 45° down the colour attraction was significantly higher (Fig. 1; van Tol, unpublished). We discovered that many glues have a hazy appearance which may therefore affect attraction of western flower thrips negatively. Davidson et al.^34^ compared different glues on only blue plates and found Stikem to be the best glue but did not connect the visual properties of the glue to more catch but more emphasizing the importance of testing one glue only when looking for colour preference. Prokopy^35^ found Stikem to be the better glue in catching apple maggot flies compared to Tangle Foot because it stayed tackier during cool weather. On the other hand, Prokopy^36^ looked at catches of apple maggot flies testing different shapes and colours thereby providing information on the spectral curve for colours with and without Stikem glue. Unnoticed he found next to red colour a high attraction to blue coloured spheres and the measurements showed shifting of colour reflectance in the presence of Stikem. With Stikem, yellow plates had more green/blue reflection and blue plates showed an increased blue and UV reflection. It is therefore possible that hazy glues affect the colour reflection of the plates and thereby causing a shift in preference for certain colours by insects.

To test our hypothesis that haze of glue has influence on colour attraction/preference for western flower thrips we compared two glues with visual different haze (whitish appearance) on differently coloured plates (blue, yellow, white and clear) under identical climatic and light controlled conditions in a wind tunnel. We validated the wind tunnel results in practice by testing blue and yellow plates with the two glues in a rose and chrysanthemum greenhouse. With a spectrophotometer we determined the change in colour composition of the plates when covered with the different glues in the wind tunnel. In both greenhouses we also measured the light entering through the glass to exclude any differences on reflection of light between the two locations.

We further analysed a number of publications on the glue types used and compared other factors such as season on colour preference of western flower thrips to determine if light intensity (brightness) next to factors such as crop type and possible learning and genetics play a role in colour preference of western flower thrips.

## Results

### Reflection pattern colour plates with glues

**Figure 2:**
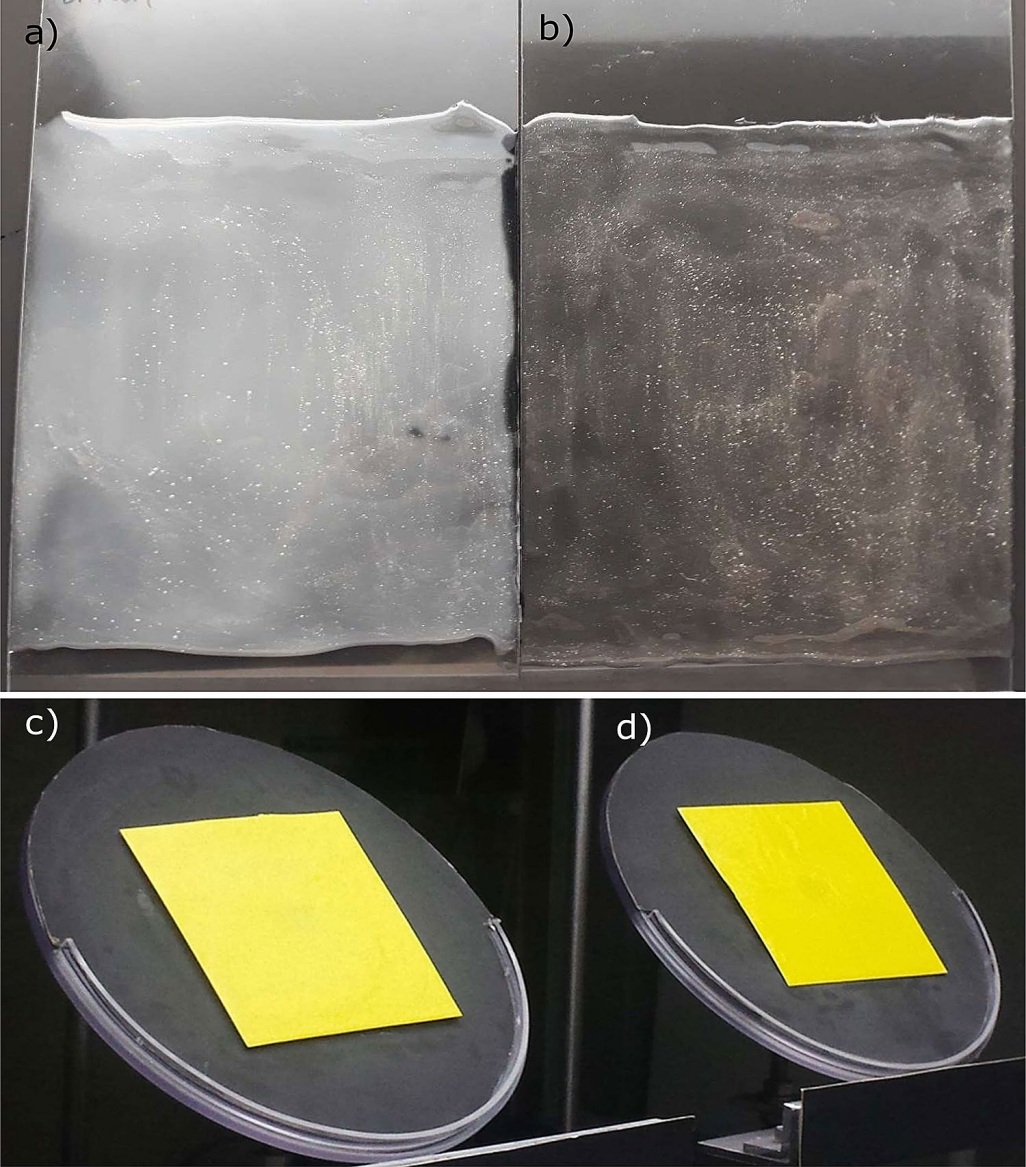
Light reflection in the wind tunnel on a clear or yellow plate with (**a**,**c**) Stikem glue or (**b**,**d**) D41 glue.

**Figure 3:**
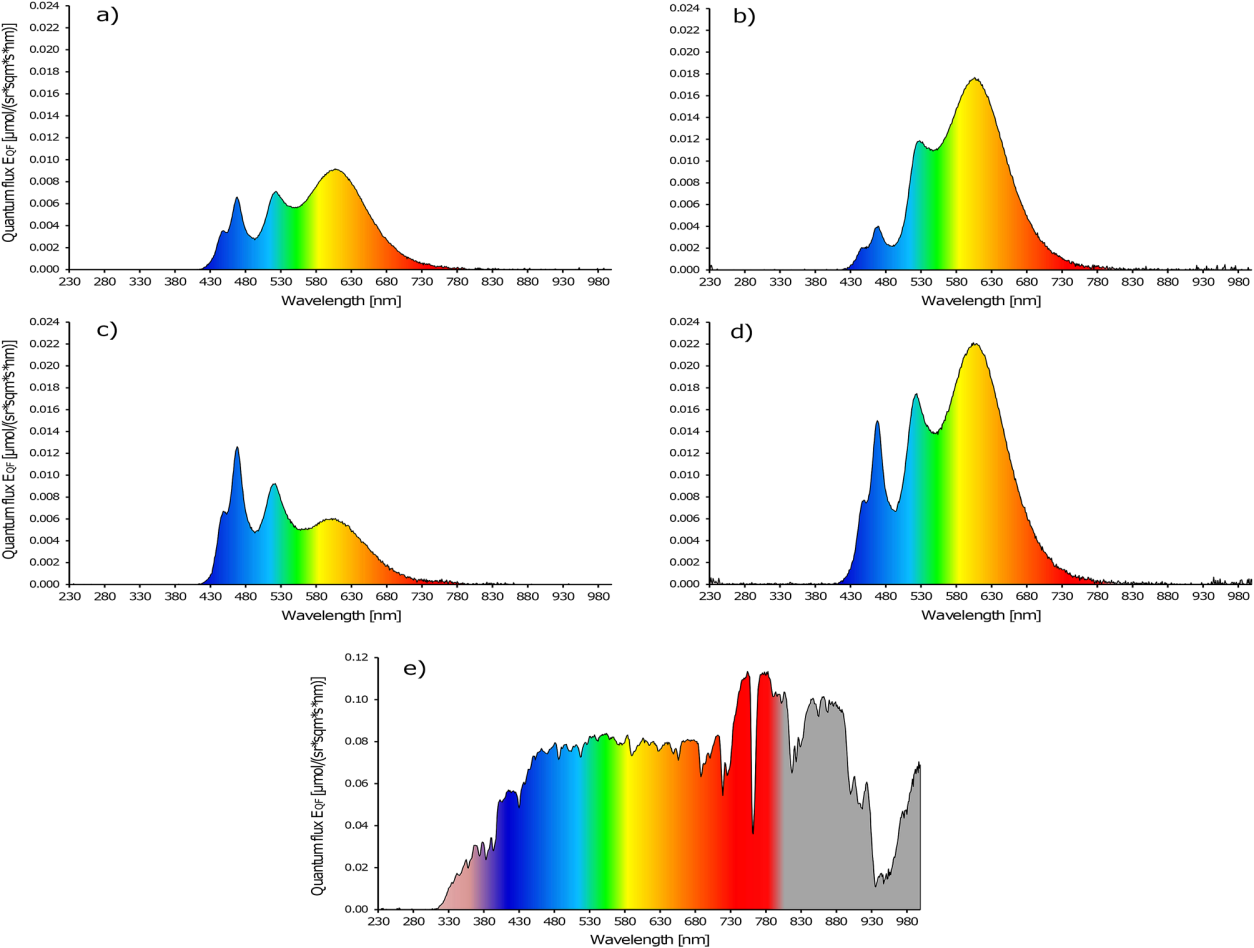
Reflection of light in the wind tunnel (**a**–**d**) on (**a**) clear plate, (**b**) yellow Horiver plate, (**c**) blue Horiver plate, (**d**) white plate with Stikem glue and in the greenhouse (**e**) measured as reflection on spectralon. Wind tunnel roof light respectively greenhouse sunlight of the thrips visible light (400–635 nm)(measured as spectralon reflection) = 3.64 respectively 17.43 µmol/(sr m^2^ s) and white plate reflection = 2.93 µmol/(sr m^2^ s). Reflection (%) of the roof light for Stikem respectively D41 on clear plate is 41.2 and 34.2, on yellow plate 65.4 and 59.8, on blue plate 44.1 and 45.3, and white plate 98.9 and 95.7.

**Figure 4:**
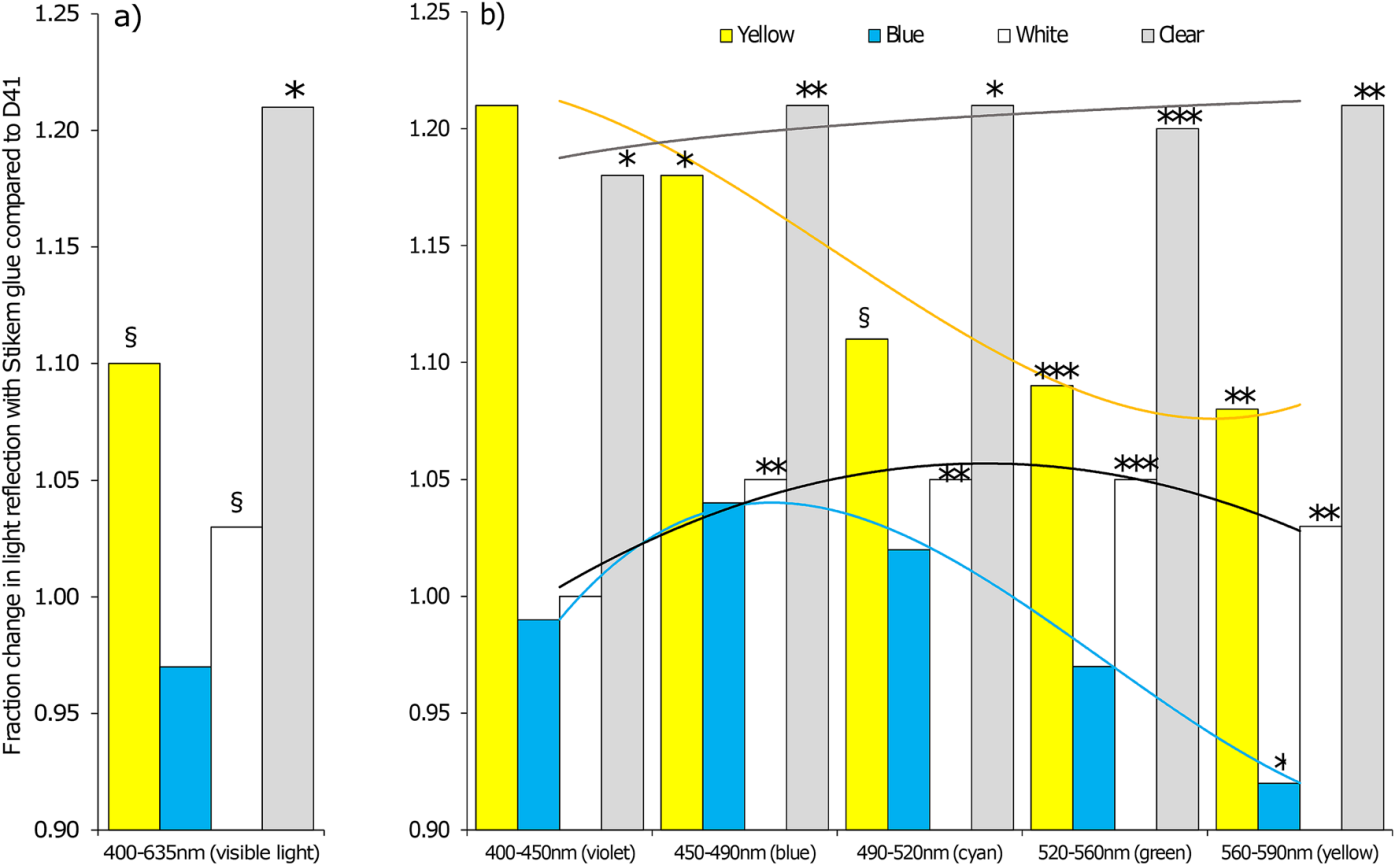
The reflection of D41 compared to Stikem glue calculated as the fraction (reflection of Stikem/D41), shown for different plate colours of (**a**) the sum of thrips visible light and, (**b**) the different light spectra (N = 3). The measurements were performed in the wind tunnel and the reflection of the underlying data (not shown) was measured in quantum flux [μmol/(m^2^ s)]. The percentage of change was considered to be significant (^§^*P* < 0.1 trend, *P < 0.05, **P < 0.01, ***P < 0.001) if the compared groups had different reflection. At significant fractions > 1, Stikem reflects more light than D41 and at < 1 it is vice versa. The statistical results were based on single *t* tests for the sum of visible lights (**a**) and a Sidak pairwise comparison of the interaction of a two-way ANOVA for glue, spectrum and interaction, for each plate colour (**b**). Test statistics, apart from symbols for significance are not shown. Trendlines for each colour plate are illustration of light reflectance shifts per wavelength group.

The change of light reflection on the coloured plates with the different glues in the wind tunnel are visible by the whitish haze in Fig. 2. The spectrophotometer measurement of coloured plates treated with Stikem are shown in Fig. 3. We did not include a picture of D41 glue as the visual differences are very small to see and are better expressed by Fig. 4. We calculated the blue (B) to green (G) ratio and the blue (B) to yellow (Y) ratio of the coloured plates for both glue types as these ratios are mentioned to play a role in colour preference of coloured LEDs (colour opponency) when both colours are mixed in different ratios. By comparing these ratios for the light entrance via the roof in the wind tunnel with the light entrance in the greenhouse we can also see if these ratios of light are substantially different. For the wind tunnel roof light B:G = 42:58 and B:Y = 45:55. The greenhouse B:G = 48:52 and B:Y = 56:44. Little difference is found for B:G ratio (6% shift to more blue and less green in the greenhouse compared to the wind tunnel) and a little larger difference is found for the B:Y ratio (11% to more blue and less yellow in the greenhouse compared to the wind tunnel). The total percentage of the thrips visible light (400 to 635 nm) for these three colours are nearly equal (wind tunnel B:G:Y = 15:21:18 and greenhouse B:G:Y = 18:19:14). The ratios of B:G reflection of Stikem and D41 glue for the yellow plates are respectively 20:80 and 18:82, the blue plates 56:44 and 54:46, the white plates 40:60 and 40:60, and the clear plates 42:58 and 41:59. The B:Y reflection of Stikem and D41 glue for the yellow plates are respectively 21:79 and 19:81, the blue plates 67:33 and 64:36, the white plates 43:57 and 43:57, and the clear plates 45:55 and 45:55. In Fig. 4 we analysed the different light colours for change in reflection. The measurements of the light reflection indicated 18% increase of blue light reflection, 9% increase of green and 8% increase of yellow light reflection from a yellow plate treated with Stikem when compared to a yellow plate treated with D41 (other colours reflected from yellow plates showed too much variation even though the percentage change was high). The sum of all visible light only showed a trend towards an increase in reflection from a Stikem glued yellow plate compared to a D41 glued yellow plate (*P* = 0.06). The reflection on the blue plate gave only small changes in light reflection whereby blue increased 4% and yellow decreased 8% of the reflected light on Stikem versus D41. For white plates there was a general increase of 3 to 5% (except violet which is near 100%) of the reflected light in the colour groups and the clear plates gave a general 20% increase of light reflected in all colours. The total brightness of the plates in the visible range increased for Stikem to D41 except for blue which was slightly less bright with Stikem compared to D41 (Fig. 4).

### Wind tunnel experiments

**Figure 5:**
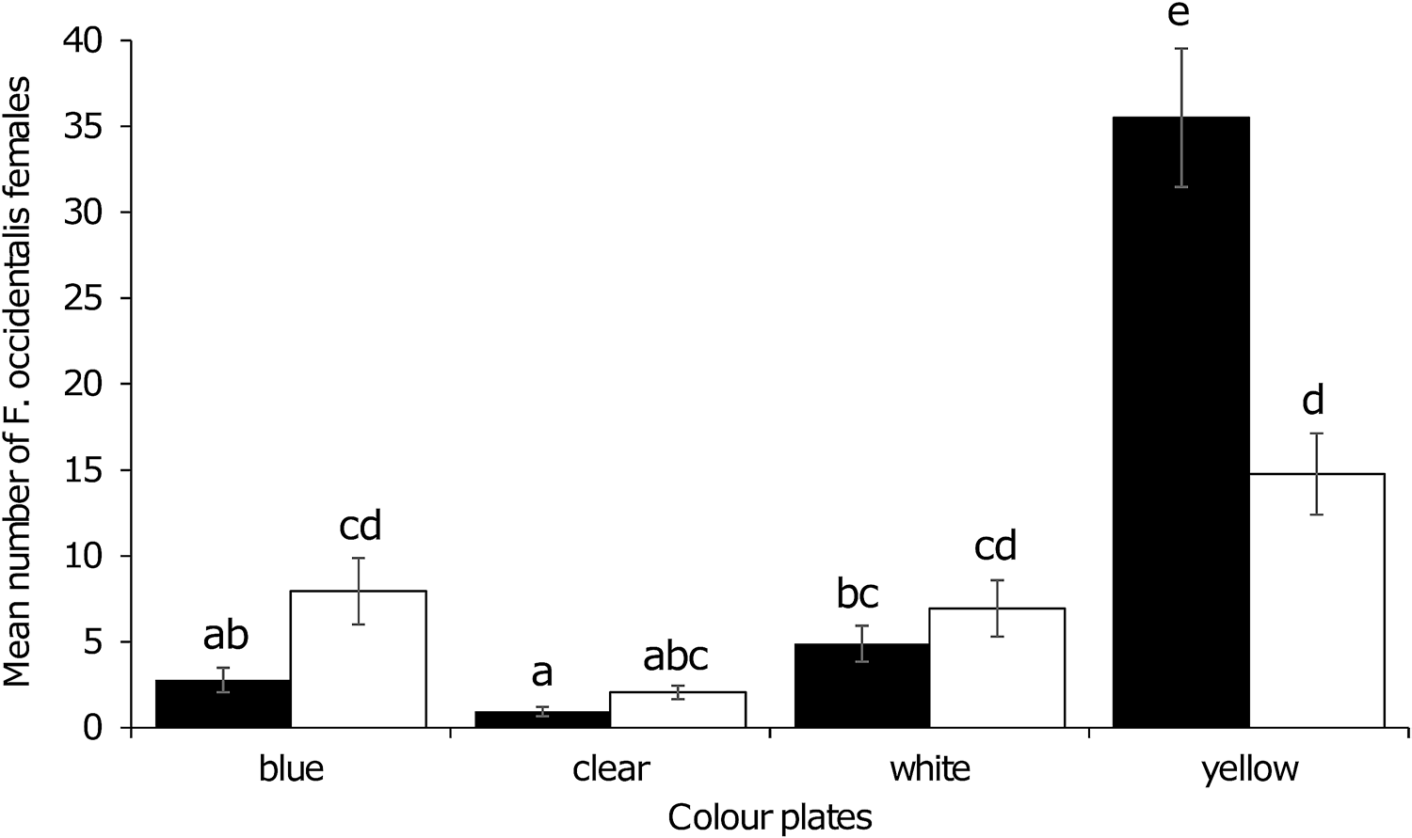
Mean number of *Frankliniella occidentalis* caught on coloured plates with Stikem glue (white bars) or D41 glue (black bars) in a wind tunnel (N = 18). Different letters (a, b, c, ….) above the bars indicate significantly different values at *P* < 0.05. Error bars represent standard error values.

The results of thrips response in the wind tunnel experiment are shown in Fig. 5. There is a significant effect of the interaction between colour and glue (Table 1, Fig. 4) on thrips catches. The number of western flower thrips was highest on yellow when using clear glue (D41) and significantly different from the other colours and glue. With diffused glue (Stikem) there was a significant decrease of attraction to yellow (58%) and a significant increase to blue (65%) compared with D41 glue. Significant differences in thrips response (except for clear plates) to the coloured plates with diffused glue were not found.

**Table 1:** Linear mixed model (LMML) for the effects of the independent variables.

	df	F value	P value
**Wind tunnel source**			
Colour	3	35.0	< 0.001
Hour	5	36.0	< 0.001
Glue	1	0.8	0.38
Colour * hour	15	2.1	0.08
Colour * glue	3	5.6	< 0.01
Glue * hour	5	0.7	0.63
Colour * glue * hour	15	1.6	0.19
**Greenhouses source**			
Crop	1	67.6	< 0.001
Colour	2	39.2	< 0.001
Glue type	1	0.004	0.95
Crop * colour	1	14.4	< 0.001
Crop * glue	1	0.004	0.95
Glue * colour	2	7.0	0.001
Crop * glue * colour	2	2.0	0.13

The applied model of estimation (REML) attributes to the number of western flower thrips caught on coloured sticky plate traps in the wind tunnel and two greenhouses (rose and chrysanthemum). The sample size is N = 144 (wind tunnel) and N = 175 (greenhouses), the value of the Akaike information criterion is 140.3 (wind tunnel) and 1703.6 (greenhouses), and the repeated covariance type of the repeated measure (h) is Unstructured (windtunnel). The number of thrips in the windtunnel data was LN (+ 1) transformed to meet the requirements of the test.

### Greenhouse experiments

**Figure 6:**
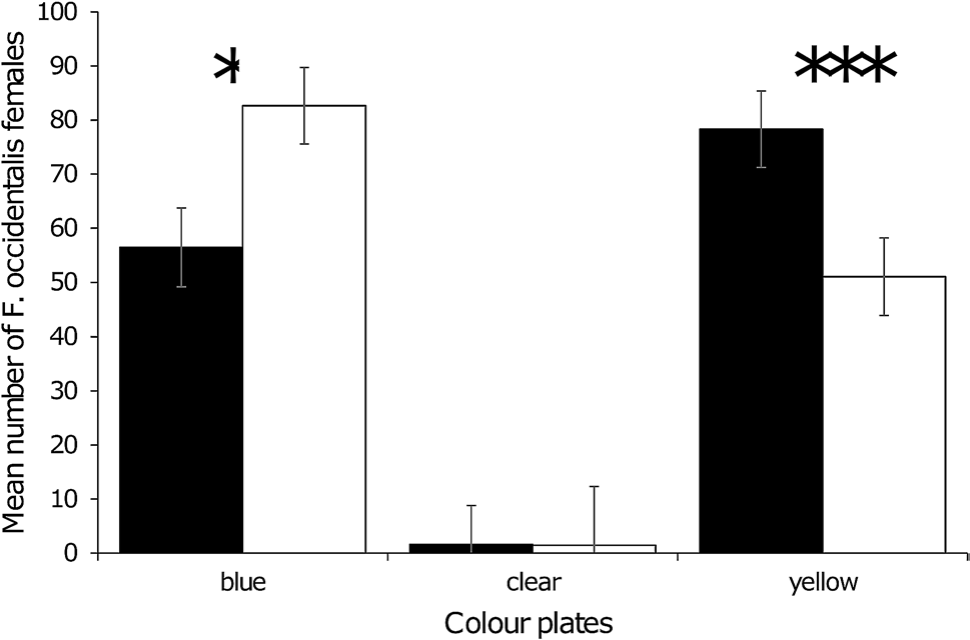
Mean number of *Frankliniella occidentalis* caught on coloured plates with Stikem glue (white bars) and D41 glue (black bars) in greenhouses (N = 14–17). Statistical result are based on normalised values. *P < 0.05, ***P < 0.001. Error bars represent standard error values.

We found in the rose greenhouse, *F. occidentalis* on 90% of blue plates and 87% of yellow plates. In the chrysanthemum greenhouse, *F. occidentalis* was found on 77% of blue plates and 91% of yellow plates. Analysis was performed on the corrected data for western flower thrips and shown in Fig. 6. In both greenhouses, we found a significant effect of the interaction between colour and glue (Table 1). In both crops there was a significant decrease in catch from yellow (35%) and increase to blue (32%) when using Stikem glue compared to D41 glue. Overall, with Stikem glue there was a preference to blue over yellow and with D41 glue a preference of yellow over blue. The light reflection in both greenhouses was similar whereby the glass of the greenhouses blocked approximately 90% of the UV-B and approximately 10% of the UV-A^37^. Other colours entered the greenhouse similar to the outside levels.

## Discussion

Coloured sticky traps are used worldwide in agriculture to determine if a pest is present. Further, these traps are used to take decisions on spraying or applying biocontrol. Recently there is more interest in using these traps for mass-trapping of insects^4,38–40^. This use leads to large amounts of plastic traps that need to be thrashed or recycled after use. Further, a high variability in effectiveness of coloured sticky traps for attraction of western flower thrips has been recorded whereby many factors are suggested to be involved as summarized by Johansen et al.^20^. This makes the optimal colour choice for mass-trapping strategy difficult. It is not clear how effective each trap and trap colour or glue is in catching western flower thrips and there are no data on how many traps are needed to be effective. Therefore, this strategy is currently using a high density of traps per hectare of greenhouse. Hence optimization of the coloured sticky traps for effectiveness can lead to precise estimates on the number of traps required per unit area. This could significantly reduce the number of traps used currently.

Most tests on determining what colour is effective is based on comparison of coloured traps whereby only in few cases the factor glue is considered as a factor of importance^34–36,41–44^. One of the least mentioned factors of influence on colour preference is glue type. There are only few reports where they looked at glue and light reflection^26,36^ and most of these researches are focusing only on more catch because of properties other than colour preference, such as stickiness and only one report on disturbance of fluorescence^45^. The influence of glue on light and colour reflectance and the corresponding effect on insect attraction was not studied.

We tested if haze of glue has influence on colour attraction/preference for western flower thrips. We compared two glues with visual different haze (whitish appearance) on differently coloured plates (blue, yellow, white and clear) in a wind tunnel and validated the wind tunnel results in practice by testing blue and yellow plates with the two glues in a rose and chrysanthemum greenhouse. We found an influence of clear versus diffused (haze) reflection of glue on change of preference for colour by western flower thrips in both wind tunnel and greenhouses. Diffused glue reduced attraction of western flower thrips to yellow compared to blue while for clear glue yellow was more attractive than blue. The change in preference cannot be accounted to the physical property of stickiness as there is a reduction in attractiveness for yellow and not for other colours when testing Stikem glue compared to D41 glue. Further the greenhouse test indicates that for the two tested plant types (rose or chrysanthemum) the plant type plays no role in the colour preference of western flower thrips.

To determine what factor in the light may lead to this change we measured the light reflectance with a spectrophotometer in the wind tunnel for all colour and glue type combinations tested. The reflection of light on clear plates for Stikem glue is on average 20% higher for all light colours than D41 (Fig. 4). On coloured plates the effects differ according to colour which can be explained by absorption of part of the diffused reflected light by the differently coloured plates tested (e.g. yellow absorbs larger part of the blue and blue larger parts of the yellow). This is confirmed by the more or less even reflection increase of 3–5% for all wavelengths on white plates with Stikem compared to D41. Yellow plates with Stikem reflect substantially more violet and blue (*P* < 0.05) and blue plates reflect less yellow and more blue (*P* < 0.05) compared to D41 glue. Each hazy wax part reflects white light in higher amounts than the surrounding plate colour where the light does not hit the hazy wax. This can be seen by the whitish yellow in Fig. 2 on yellow plates with Stikem versus the clear yellow plates with D41. It is unclear why the white reflection of wax in the glue leads to rejection of yellow but not blue. Colour constancy seems unlikely to play a role. Colour constancy is known and described for most insects and reviewed by Chittka et al.^46^. Colour constancy is described as the perceptual phenomenon that the colour of an object appears largely unchanged, even if the spectral composition of the illuminating light changes. It means that the insect can still recognize a colour under different spectral conditions. In our experiments, however, not the spectral conditions change but the reflection of parts of the surface due to the wax changes and this leads to a reduced attraction to yellow. Another cause of preference change could be the colour opponency theory^47–49^. It suggests that when blue and yellow wavelengths are presented together, the excitatory response of colour-opponent neurons is partially or completely suppressed. Blue (B) and yellow (Y) together will be seen as blue by the eyes. Although the B:Y ratios changes only slightly between Stikem and D41 on yellow plates (see results: 2%) it has been shown that small changes in composition can lead to major shifts in behaviour^30^. The recently published “blue-green opponency” of western flower thrips^29^ is a colour opponency that may play a role even though our thrips strain responds in general better to yellow, which is considered a bright supernatural green to insects. While Stukenberg et al.^29^ showed that blue and green LEDs were attractive, addition of green LED to blue LED decreased attraction compared to its single components. We measured increased reflection of blue and green on yellow sticky plates with Stikem and decreased reflection of green on blue plates with Stikem compared to D41 (Fig. 4). These shifts in colour change are significant and although seemingly small may have a large effect on behaviour as shown for whiteflies^30^. Our measurements of light in the greenhouses versus the wind tunnel indicate near identical percentages of blue (B), green (G) and yellow (Y) relative to the total thrips visible light. These percentages are based on the wavelength categories as defined. In both wind tunnel and greenhouses the fractions of blue and green of the light entering is only slightly different (B:G wind tunnel = 42:58, greenhouses 49:51) and reflection between the two tested glues on yellow plates in the wind tunnel show that the ratio of B:G only shifts 2% from 20:80 for Stikem to 18:82 for D41. Stukenberg et al.^29^ showed that ratios of B:G applied as LED colours of 50:50 and even 25:75 are reducing attractiveness strongly. It is still unclear if the total increase of blue and green on yellow plates with Stikem initiates this opponency mechanism. From our work with paint colours versus LED pure colours as performed by others it remains unclear if the blue-green opponency plays a role since brightness aspects (unequal intensity reflection between colours on sticky plates compared to equal intensity of pure colours by LEDs) may be involved in attraction as well. Complicating is the result by van Tol et al.^37^ where fluorescing white increases blue reflection leading to more attraction of *F. occidentalis*. If blue-green opponency plays a role it can be even in white coloured material with a shift of ration of B:G to higher values. The white reflecting light of the wax particles in the glue are most certainly playing a role in the disturbed attraction to yellow, as the high values of change on all colours by clear plates with the two glues (whitish appearance) (Fig. 4) show.

There are only few publications where Stikem glue has been tested. Vernon and Gillespie^50,51^ tested in summer different coloured plates with Stikem and found blue to be more attractive or equally attractive as yellow for western flower thrips. Further Stikem was also tested in a flowering apple orchard in spring whereby blue and white were equally attractive and yellow was performing poorly^27^. In this case white flowers and white plates may have led to an adaptive behaviour to white which is in most other papers mentioned as a minor attractive colour for western flower thrips. Blackmer et al.^26^ mentions white and blue to be more attractive than yellow and even clear plates attracted substantial thrips numbers indicating the strong effect glue haze has on colour preference. They were using Pestick glue which appears to have a strong haze like Stikem as the reflection measurements they performed showed. Yudin et al.^28^ also found white to be more attractive than blue or yellow. It is unknown if the used glue (Tack Trap with poly isobutylene as glue) has any strong haze. In a wind tunnel trial different glues, including Stikem, were tested on attractiveness of western flower thrips^34^. It appeared that Stikem was catching most thrips. Unfortunately, this was done only with blue plates and no conclusion was drawn in relation to increased visual response of thrips explaining the more catch. In studies conducted with apple maggot fly, they used Stikem and concluded that they caught more apple maggots than with Tree Tanglefoot because of the tackiness differences between the two glues as caused by cooler weather^35^.

Where glue and haze of glue play a role in preference, other factors like season, learning and genetics may play a role as well. Attraction to white of western flower thrips seems rare and there are few publications with either strong attraction (equal or better than blue)^26–28^ or no attraction at all to white as our results show but also others^19,52^. Dichromatic colour space only has two dimensions, hue, saturation and brightness are not independent^53^. As such around 480 nm (blue) light appears the same to a dichromat insect as broad-spectrum white light. It is still unclear if thrips is trichromatic or dichromatic. Yang et al.^54^ performed an interesting comparison to blue and yellow LED preference whereby they varied the intensity of the light from low to high (20 to 100 lx). At low intensity (40 lx) blue was more attractive than yellow (32.4% to blue versus 20.7% to yellow) whereas at 80 lx yellow was more attractive than blue (41.4% to yellow versus 16.5% to blue). Although it is assumed that photoreceptors respond maximal when triggered by a certain level of photons^55,56^ this result suggests a negative feedback at higher photon levels of blue which may be connected more to complex colour vision pathways in the optic lobes^55,57,58^. If this is true than blue reception is more sensitive than yellow at a lower photon level. This may correlate to a number of publications where blue appears to be more attractive than yellow as they were performed in winter, spring or autumn^4,16,18–20,22,23^. Further there are two more publications where they followed trapping of western flower thrips over the season for blue and yellow whereby this result is confirmed. Yudin et al.^28^ found an equal attraction to blue and yellow in the period October to March and more attraction to yellow than blue in April (65% on yellow versus 26% to blue) and May (63% to yellow and 13% to blue). Rőth et al.^23^ found blue to be more attractive in December (20.8 thrips per blue trap versus 0 per yellow trap) and equal numbers in March (26.6 thrips per blue trap versus 31.6 thrips per yellow trap). He found especially in winter a much higher catch with fluorescent yellow (117.4 thrips per trap) but this can be attributed to the much larger light reflection in the blue/green area (approximately 50% more light reflectance in the area 480–510 nm) than the yellow area (approximately 20% more light reflectance in the area 560–590 nm) compared to reflection on the blue and yellow plates. Again, this preference has disappeared in March where blue, yellow and fluorescent yellow perform equally well. Excluding all results with the known hazy Stikem and Pestick glue and lab experiments and only including literature in the field and greenhouse with blue and yellow leave 13 publications to judge colour preference. Of them 7 publications were performed in winter, spring or autumn^4,16,18–20,22,23^ and 6 in summer^17,21,24,25,28,59^. In 100% of the winter/spring/autumn publications blue is more attractive than yellow in trapping western flower thrips^4,16,18–20,22,23^ and no publications with yellow better than blue. In contrast tests performed in summer (6) show 50% yellow better than blue^24,25,28^ and 33% have blue better^17,21^ or equal (17%) to yellow^59^. The location of the trials may also influence the different results as the angle of the sun does not differ as much for both seasons near the equator. There is however only one trial near the equator (Kenya) where blue and yellow are equally well performing in summer and winter^59^ and several subtropical locations like California, Arizona, Hawaii and Australia where the winter trials versus summer trials are more or less showing the same results as for the more northern regions such as Norway, Hungary, Germany and Washington State (USA) where mostly the winter trials show a preference for blue over yellow. This little survey next to the seasonal effects found in some publications^23,28^ are indicating a seasonal response to blue and yellow, correlated to the intensity of light in winter versus summer in accordance with the lower brightness preference to blue Yang et al.^54^.

Effect of background crop or flowers may also have a large influence on colour preference. Gillespie and Vernon^27^ showed equal attraction of western flower thrips to blue and white in a flowering apple orchard. Hereby the olfactory-visual interaction may have played a substantial role. In lab trials with a black background usually yellow is the preferred colour and in greenhouses blue is the preferred colour^60^. In lab experiments with different foreground–background coloured plates the influence of background colour on attraction has been shown^61^. While differences between lab and greenhouse can be correlated to light intensity, background colours and environmental conditions, these differences are much harder to compare in the field. For different crops not only colour but also different olfactory and climatic conditions influence thrips behaviour. As such the preference for blue over yellow in French bean crop^62^ versus no preference to yellow or blue in tomato^59^ could be attributed to olfactory factors as well as crop colour.

Where the strong response to blue and yellow is substantially changed due to the visual aspects of the glue on the plates other factors like brightness (season) and possible learning^27^ or genetics^28^ play a role in blue/yellow/white preference of the western flower thrips. We further investigate the role of genetics and season on colour preference in follow-up studies.

## Material and methods

### Reflection pattern colour plates with glues

The reflection of the used yellow and blue plates from Koppert B.V. (Horiver), as well as the white (source unknown) and clear (Pherobank) plates is determined with a spectrophotometer [Specbos 1211 UV (Jeti Techn. Instr. GmbH)] in the wind tunnel with the respective glues of D41 and Stikem on them (N = 3). Measurement of the plates with the different glues provide small differences in specular versus diffused reflection of the light due to the small diameter of the beam that is measuring only a fraction of the reflected light from the plates. Despite this, we measured radiance to determine the relative light reflection of Stikem versus D41 (Fig. 4) to determine the change in reflection and colour composition occurring when using different glues. Each plate is positioned 45° to the roof and the light measured by a horizontally placed spectrophotometer at a distance of 20 cm from the center of the plates. Each plate is 10 × 10 cm sized and placed on a black paper as a background. The reflection pattern of the different plates reveals differences in the total amount of light reflected (brightness) and the colour composition of the light reflected by the plates. As a reference we measured the light reflection of a Spectralon plate. Spectralon reflects 99.9% of all light colours and is as such a qualitative reference for the absolute light that enters the wind tunnel. Measurement of the black background alone did not reveal any light that could be measured by the spectrophotometer.

### Thrips rearing

Western flower thrips (*Frankliniella occidentalis*) is maintained at 25 °C and 60–70% relative humidity (RH) with a 16:8 light:dark period in potted flowering chrysanthemum (*Chrysanthemum morifolium* Ramat.) (syn. *Dendranthema grandiflora* Tzelev). Female western flower thrips of unknown age and mating status were used for all trials. For each experiment 100 female thrips are collected from the rearing and kept in a clear plastic box (diameter 8 cm, height 6 cm) covered by a Parafilm (Bemis, PM-996, USA) layer until release in the wind tunnel. Pre-testing showed no effect of mixed age females versus same aged females and starvation of 0.5–6 h indicated that after 1-h starvation the maximum response to the visual objects was reached.

### Climate and light settings wind tunnel

We conditioned the wind speed, light, relative humidity and temperature in the wind tunnel based on optimal data for flight of thrips^63^. Wind speed was set to 3 cm/s, temperature to 26 °C and relative humidity to 70%. Roof illumination for the wind tunnel was adjusted to imitate the wavelength spectra of natural sunlight as close as was feasible with commercially available LEDs (LED-strip—Full-color RGB + Warm White—24 V High Power Protected 5050, LuxaLight, NL) (Fig. 3). Emission spectra of the LEDs and roof illumination were measured and adjusted inside the wind tunnel using a broadband spectroradiometer Specbos 1211UV (JETI Technische Instrumente GmbH, Germany). We limited ourselves to the visible light (400–720 nm). We measured the quantum flux light of the roof entering the wind tunnel as reflection on a spectralon plate. We measured a reflection value of 3.64 µmol/(sr m^2^ s) for the thrips visible light (400–635 nm). Pre-testing in advance on the influence of adding UV-A (365 nm) to the roof light revealed no different responses on colour choice by western flower thrips. We also excluded infrared which is outside the vision range of western flower thrips. Western flower thrips has a vision range from approximately 355–635 nm^15,64^. The wind tunnel is length × width × height = 300 × 120 × 80 cm of which we lighted only the central part (100 × 120 × 80 cm) which is the test arena. The central roof part was covered by a UV transmitting transparent polyethylene diffusing sheet on the top (Suncover Nectarine C-980, Ginegar Plastic Products Ltd., Israel) to limit landing of the thrips on the roof. The other parts were covered by black fabric preventing light entering the wind tunnel.

### Wind tunnel experiment

The release box of thrips was placed on a platform at a distance of 60 cm from the center of the visual objects on a platform at a height of 15 cm above the wind tunnel floor. The visual objects were placed at an angle of 45° on a height of 40 cm relative to the floor of the wind tunnel on a circular platform covered with black paper (Fig. 2). Each visual object was cut from unglued Koppert plates (Horiver yellow and blue: 10 × 10 cm) or clear plastic (Pherobank) and white plates that were glued with either D41 or Stikem. Adult females (distinguished from males by their larger size) of unknown age and mating status were collected from the colony using an aspirator and then transferred to a transparent plastic box ("release container") by sedation with CO_2_ for 15–20 s. Each trial used 100 insects. The release container was covered with parafilm and placed on the release platform to acclimate the thrips before starting the experiments. After exactly one hour of acclimation, the insects were released by removing the parafilm from the release box with minimal physical disturbance into the tunnel. Thrips were monitored each hour (with a binocular from outside the wind tunnel) for 6 h total after which the experiment ended. We counted both the thrips that remained in the release box and the thrips that landed on the visual objects for each hour. Each treatment was repeated 18 times.

### Greenhouse experiment

We tested yellow and blue plates from Koppert (Horiver, size 10 × 25 cm) that were free of glue and glued them with either D41 or Stikem. We placed the different plates in two greenhouses with different crops (chrysanthemum and rose). We measured the sunlight spectrum as reflection on a spectralon plate, entering the greenhouse in June with the same spectrophotometer as used in the wind tunnel (Fig. 3e). We measured a reflection value of 17.43 µmol/(sr m^2^ s) for the thrips visible light (400–635 nm). Sticky plates were placed at 20 m distance from each other and 2 traps per date for each treatment were tested. Treatments were repeated weekly for 4 consecutive weeks in June and July whereby we refreshed the plates each week. In total 14–17 plates for each treatment were tested. We counted the number of thrips and took subsamples from the different plates to determine thrips species composition. From each date we subsampled 1 plate of each colour per treatment to determine the composition of the thrips species. To do this thrips were removed from the sticky traps using De-Solv-it (RCR International, Victoria, Australia). All thrips were counted and from each trap a maximum of 50 randomly selected thrips were mounted on to slides for species identification. If the total number of thrips per trap was lower than 50, all thrips were mounted for identification. All thrips were identified under 100× magnification^65^. Numbers of thrips were corrected according to the ratio of the species found on the plates before statistical analysis. We measured the light in both greenhouses with the spectrophotometer to determine if any differences in incoming light exist between the two companies.

### Statistical analysis

All statistics have been performed using statistical package IBM SPSS statistics version 25.

### Light reflection

We used four independent student’s T tests for the differences of total light reflection (400–635 nm) between glues for each plate colour (N = 3). We used four two-way ANOVA’s the differences of the light colour reflection between glues, and the interaction, for each plate colour, with a Sidak post hoc test for the differences between glues within light colour. When the interaction was not significant, but both other factors where, we assumed all changes as calculated within Fig. 4 for that plate colour where significant.

### Wind tunnel experiments

We used a linear mixed model for the number of thrips caught on the trap. The number of thrips was LN (+ 1) transformed, to meet the assumptions for normality based on the results of the Shapiro–Wilk test for normality on the model residuals and visual inspection of the frequency histogram. The number of trips was modeled, as a function of glue type, colour of the trap, light (with the addition of different levels of the colour of the light), hour (each experimental run ran for 6 h where every hour the thrips on the traps were counted), and multiple 2-way and 3-way interactions between these variables. As the number of thrips accumulated on the traps, measurements between hours were not independent. Hour was added as a repeated measure with the date of the experiment (maximum of one experiment was done each day) as subject (covariance type was unstructured). There were no differences found on the take-off numbers from the release platform. After 6 h near all thrips had left the box. There was therefore no number correction of thrips related to the number still in the box. The best fitting model was selected by choosing the covariance matrix for the repeated measures with the lowest Akaike information criterion (AIC) value. The Hurvich and Tsai's Criterion (AICc) value was used at low sample sizes (N < 50). Additionally, the model restricted maximum likelihood (REML) or maximum likelihood (ML) with the lowest AIC(c) value used. To test the main effects of the significant interactions, Sidak’s pairwise comparisons were performed. Results of the analysis are shown in Table 1.

### Greenhouse experiments

Analysis of data was performed after correction of the thrips data for western flower thrips. Several linear mixed models were used. For all linear mixed models, the assumptions of normality were met (without transformation) based on the results of the Shapiro–Wilk test for normality on the model residuals and visual inspection of the frequency histogram. The best fitting model was selected by choosing the covariance matrix for the repeated measures with the lowest Akaike information criterion (AIC) value. We tested the residuals of the model for normality using the Komogorov–Smirnova test, with a *P* > 0.20 threshold in combination with a visual inspection of the residuals’ histogram. The Hurvich and Tsai's Criterion (AICc) value was used at low sample sizes (N < 50). Additionally, the model restricted maximum likelihood (REML) or maximum likelihood (ML) with the lowest AIC(c) value used. To test the main effects of the significant interactions, Sidak’s pairwise comparisons were performed. Results of the analysis are shown in Table 1.

## Data Availability

The datasets generated during and/or analysed during the current study are available from the corresponding author on reasonable request.
